# Draft Genome Sequence of Linezolid-Resistant *Enterococcus faecalis* Clinical Isolate HS0914

**DOI:** 10.1128/genomeA.00782-14

**Published:** 2014-08-07

**Authors:** Dongfang Lin, Yan Guo, Chunhui Chen, Yingjie Fuzhu, Shuxin Xiao, Demei Zhu, Minggui Wang, Xiaogang Xu

**Affiliations:** a Institute of Antibiotics, Huashan Hospital, Fudan University, Shanghai, People’s Republic of China; b Key Laboratory of Clinical Pharmacology of Antibiotics, National Health and Family Planning Commission of the People's Republic of China, Shanghai, People’s Republic of China

## Abstract

We report the draft genome sequence of linezolid-resistant *Enterococcus faecalis* strain HS-0914 isolated from a teaching hospital in Shanghai, China. The draft genome sequence is composed of 61 contigs for 2,816,079 bp. Ribosomal RNA mutations and *cfr*, which mediates linezolid resistance, are not present.

## GENOME ANNOUNCEMENT

*Enterococcus faecalis* is one of the most common causes of opportunistic infections. *E. faecalis* HS0914 was isolated from a urine sample collected from a patient at a teaching hospital in Shanghai, China. The clinical isolate was resistant to linezolid (MIC 32 mg/liter), although the patient had never been exposed to it. Five point mutations in the 23S rRNA (G2576T, U2500, G2447UA, C254U, and G2603U) and ribosomal protein (L3 and L4) genes could lead to linezolid resistance in Gram-positive cocci (1–3). The multidrug-resistant gene, *cfr*, could also result in linezolid resistance (4). However, neither the mutations previous reported nor the *cfr* gene was found in the linezolid-resistant clinical isolate HS0914 by PCR and Sanger sequencing. Whole-genome sequencing of the linezolid-resistant isolate could reveal novel mechanisms of linezolid resistance. In this paper, we describe the draft genome sequence of the linezolid-resistant isolate HS0914.

Genomic DNA was prepared using the QIAamp DNA MiniKit (Qiagen). Genome sequencing was performed using Illumina HiSeq 2000 technology (Illumina, Inc.). A total of 38,235,708 paired-end reads (insert size 300 bp) were obtained. BWA was used to filter the raw data for high quality (raw data: 3,861,806,508 bp, clean data: 3,670,009,615 bp) (5). Adaptor-free reads were used for assembly with SOAPdenovo (v1.05) (6). The final assembly contains 61 contigs, with a total size of 2,816,079 bp and an *N*_50_ contig length of 105,150 bp. The lengths of the contigs ranged from 236 bp to 452,383 bp, with a G+C mol% of 37.3%. The DNA sequences were annotated using GeneMark.hmm for Prokaryotes (7). The draft genome contains 3,023 genes, with 2,963 protein-coding genes and 63 structural RNAs.

### Nucleotide sequence accession numbers.

This whole-genome shotgun project has been deposited at DDBJ/EMBL/GenBank under the accession no. JPDQ00000000. The version described in this paper is version JPDQ01000000.

## References

[1] Long KS Vester B 2012 Resistance to linezolid caused by modifications at its binding site on the ribosome. Antimicrob. Agents Chemother. 56:603–612. 10.1128/AAC.05702-11 22143525 PMC3264260

[2] Locke JB Hilgers M Shaw KJ 2009 Mutations in ribosomal protein L3 are associated with oxazolidinone resistance in staphylococci of clinical origin. Antimicrob. Agents Chemother. 53:5275–5278. 10.1128/AAC.01032-09 19805557 PMC2786331

[3] Wolter N Smith AM Farrell DJ Schaffner W Moore M Whitney CG Jorgensen JH Klugman KP 2005 Novel mechanism of resistance to oxazolidinones, macrolides, and chloramphenicol in ribosomal protein L4 of the pneumococcus. Antimicrob. Agents Chemother. 49:3554–3557. 10.1128/AAC.49.8.3554-3557.2005 16048983 PMC1196237

[4] Mendes RE Deshpande LM Castanheira M DiPersio J Saubolle MA Jones RN 2008 First report of *cfr*-mediated resistance to linezolid in human staphylococcal clinical isolates recovered in the United States. Antimicrob. Agents Chemother. 52:2244–2246. 10.1128/AAC.00231-08 18391032 PMC2415768

[5] Li H Durbin R 2009 Fast and accurate short read alignment with Burrows-Wheeler transform. Bioinformatics 25:1754–1760. 10.1093/bioinformatics/btp324 19451168 PMC2705234

[6] Li R Zhu H Ruan J Qian W Fang X Shi Z Li Y Li S Shan G Kristiansen K Li S Yang H Wang J Wang J 2010 De novo assembly of human genomes with massively parallel short read sequencing. Genome Res. 20:265–272. 10.1101/gr.097261.109 20019144 PMC2813482

[7] Besemer J Lomsadze A Borodovsky M 2001 GeneMarkS: a self-training method for prediction of gene starts in microbial genomes. Implications for finding sequence motifs in regulatory regions. Nucleic Acids Res. 29:2607–2618. 10.1093/nar/29.12.2607 11410670 PMC55746

